# Neuropeptides encoded by the genomes of the Akoya pearl oyster *Pinctata fucata* and Pacific oyster *Crassostrea gigas:* a bioinformatic and peptidomic survey

**DOI:** 10.1186/1471-2164-15-840

**Published:** 2014-10-02

**Authors:** Michael J Stewart, Pascal Favrel, Bronwyn A Rotgans, Tianfang Wang, Min Zhao, Manzar Sohail, Wayne A O’Connor, Abigail Elizur, Joel Henry, Scott F Cummins

**Affiliations:** School of Science and Education, Genecology Research Center, University of the Sunshine Coast, Maroochydore DC, Queensland 4558 Australia; Université de Caen Basse-Normandie, Biologie des ORganismes et Ecosystèmes Aquatiques (BOREA), Caen, 14032 France; CNRS UMR 7208, BOREA, Caen, France; Port Stephens Fisheries Institute, Locked Bag 1, Nelson Bay, New South Wales, 2315 Australia

**Keywords:** *Pinctada fucata*, *Crassostrea gigas*, Molluscs, Circular dichroism, Egg-laying hormone, Feed circuit activating peptide, Gonadotropin-releasing hormone, High-performance liquid chromatography, Mass spectrometry, Neuropeptides

## Abstract

**Background:**

Oysters impart significant socio-ecological benefits from primary production of food supply, to estuarine ecosystems via reduction of water column nutrients, plankton and seston biomass. Little though is known at the molecular level of what genes are responsible for how oysters reproduce, filter nutrients, survive stressful physiological events and form reef communities. Neuropeptides represent a diverse class of chemical messengers, instrumental in orchestrating these complex physiological events in other species.

**Results:**

By a combination of *in silico* data mining and peptide analysis of ganglia, 74 putative neuropeptide genes were identified from genome and transcriptome databases of the Akoya pearl oyster, *Pinctata fucata* and the Pacific oyster, *Crassostrea gigas*, encoding precursors for over 300 predicted bioactive peptide products, including three newly identified neuropeptide precursors PFGx8amide, RxIamide and Wx3Yamide. Our findings also include a gene for the gonadotropin-releasing hormone (GnRH) and two egg-laying hormones (ELH) which were identified from both oysters. Multiple sequence alignments and phylogenetic analysis supports similar global organization of these mature peptides. Computer-based peptide modeling of the molecular tertiary structures of ELH highlights the structural homologies within ELH family, which may facilitate ELH activity leading to the release of gametes.

**Conclusion:**

Our analysis demonstrates that oysters possess conserved molluscan neuropeptide domains and overall precursor organization whilst highlighting many previously unrecognized bivalve idiosyncrasies. This genomic analysis provides a solid foundation from which further studies aimed at the functional characterization of these molluscan neuropeptides can be conducted to further stimulate advances in understanding the ecology and cultivation of oysters.

**Electronic supplementary material:**

The online version of this article (doi:10.1186/1471-2164-15-840) contains supplementary material, which is available to authorized users.

## Background

Neuropeptides encompass a diverse class of cell signaling molecules that are produced and released from neurons through a regulated secretory pathway [1]. They may function as hormones, transmitters and modulators; as modulators of neuronal activity, neuropeptides contribute to the generation of different outputs from the same neuronal circuit in a context-dependent manner [2], or organize complex motor functions [3]. Neuropeptides that act as hormones are released into the haemolymph via a network of neurohemal organs, upon which they regulate various states of physiology, including growth, metabolism, and reproduction [4].

In general, neuropeptides are generated from an immature precursor that contain an N-terminal signal sequence and single or multiple copies of bioactive peptide [5]. Mature bioactive peptides are often short with low in molecular weights (<10 kDa), the shortest and smallest being dipeptides [6, 7]. Within the secretory apparatus, proteases cleave the precursor at mono- or dibasic cleavage sites [8], after which mature peptides are often further modified through post-translational modifications [9]. Conventional methods of neuropeptide characterization have involved their purification directly from neural-associated tissues in conjunction with the analysis of corresponding gene expression. Identification of cross-species neuropeptide conservation has typically relied on the use of antibody probes that bind to homologs. However, given the relative ease and affordability of genomics and mass spectrometry, the near full neuropeptide repertoire of several species has been revealed [10], even within non-model animal species [11].

One mollusc in which genomics has helped to reveal the extent of the neuropeptidome is the owl limpet, *Lottia gigantea. Lottia* is a marine gastropod that has emerged as a molluscan genome model following the recent sequencing of its relatively small genome. Data mining of the *L. gigantea* genome has revealed around 59 genes that encode for putative neuropeptides [12], most of which had been previously characterized or identified through functional testing or descriptively identified (i.e. immunohistochemistry) in other molluscs, insects or annelids. Examples of these include the tetrapeptides Ala-Pro-Gly-Trp-NH_2_ (APGWamide) and Phe-Met-Arg-Phe-NH_2_ (FMRFamide), as well as the egg laying hormone (ELH) and gonadotropin-releasing hormone (GnRH) [12]. While most genes encoding neuropeptides have not been identified in molluscs, some that have, do share distinct homology with *Drosophila*, such as the putative proctolin homolog PKYMDT and allatostatin C, which are believed to be derived from neuropeptides with an early origin from either eumetazoan or bilaterian ancestors [2, 10].

Most research in oysters has been devoted to understanding their widespread ecological impacts, nutrient processing, nutrition, larval settlement, and environmental factors that modulate spawning frequency and distribution. However, little is known of the metabolic neuropeptides that regulate these processes. Insight into this area has the potential to be either exploited for advances in oyster culture, or for controlling and understanding their natural biological processes which contribute to their invasiveness. Recently, genome sequence assemblies and annotations became available for *Pinctata fucata* and *Crassostrea gigas,* providing an excellent opportunity to characterize the repertoire of oyster neuropeptides [13, 14].

The Akoya pearl oyster *Pinctata fucata*, are wide spread and can form dense populations, but are cultured primarily for their ability to produce pearls [15]. The *P. fucata* draft genome version 1.1 (approximately 40x coverage) became available in 2012 predicting 23,257 complete gene models and includes genes associated with shell biomineralization [13], as well as reproduction-related genes involved in the process of germ cell migration; *vasa*, *nanos*, oocyte maturation, and spawning. This includes 5-hydroxytryptamine, vitellogenin and estrogen receptors [16]. On the other hand in the same year analysis of the highly polymorphic *C. gigas* genome and transcriptomes revealed an extensive set of genes that provides a rare glimpse of how *C. gigas* respond to environmental stress, and adapt to near environments, as well as giving insight perspective into the molecular mechanism of shell formation, development and reproduction [14].

In this study, we interrogated the genomes and transcriptomes of *P. fucata* and *C. gigas* to identify neuropeptide genes. To help support gene predictions, we performed comparative analysis and peptidomic investigation of *C. gigas* ganglia. Among those neuropeptides identified are those known to be involved in molluscan reproduction (e.g. APGWamide, egg-laying hormone and gonadotropin-releasing hormone) and growth (e.g. FMRFamide). This study provides a foundation for the experimental analysis of neuropeptides in oysters, which can be used to increase focus on the loss of associated ecosystem and food supply services that oysters contribute to the environment and human well-being.

## Results and discussion

**Figure 1:**
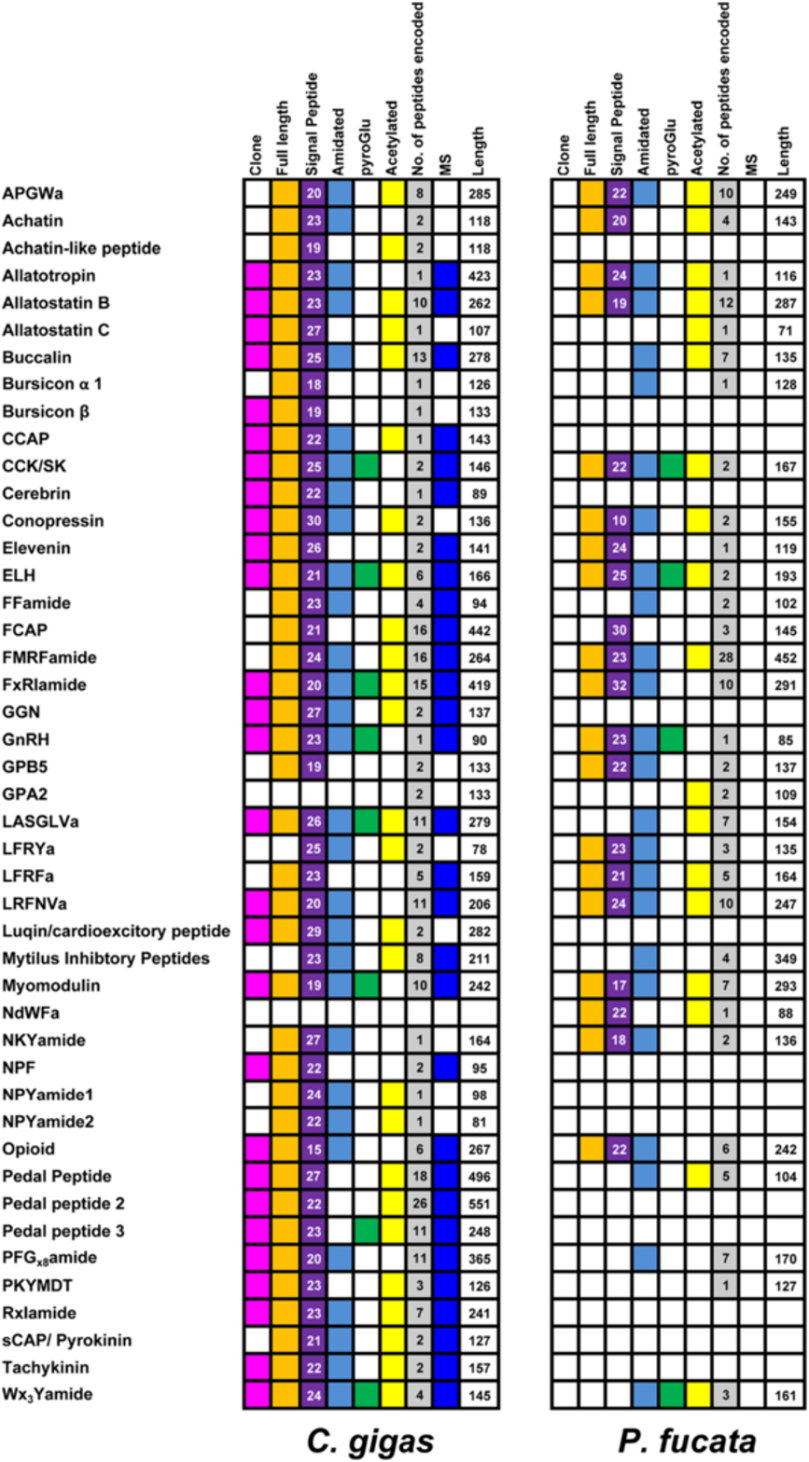
**Summary of identified genes encoding putative full-length or partial-length neuropeptide precursors from the** ***Pinctada fucata*** **and** ***Crassostrea gigas*** **genome, and transcriptome databases** ***.*** For each neuropeptide we highlight whether a cDNA clone, a full-length ORF sequence, and MS evidence are available. We also indicate the numbers of peptides that are cleaved from the mature neuropeptide precursor, if they are amidated, acetylated, or pyroglutaminated peptides, and if a leader signal peptide is encoded.

We have identified genes encoding putative full-length or partial-length neuropeptide precursors from the *P. fucata* and *C. gigas* genome, and transcriptome databases for *C. gigas* (Figure 1 and Additional file 1). Numerous peptides are released from these precursors, some of which were confirmed from *C. gigas* neural tissue by liquid chromatography-tandem mass spectrometry (LC-MS/MS) analysis (Additional files 2, 3 and 4). For some of the neuropeptides, defined roles in reproduction and growth have been established and will be discussed in the context of the newly identified oyster sequences. Database accession numbers for sequences used in this study can be found in Additional files 2 and 5.

### APGWamide and FMRFamide

**Figure 2:**
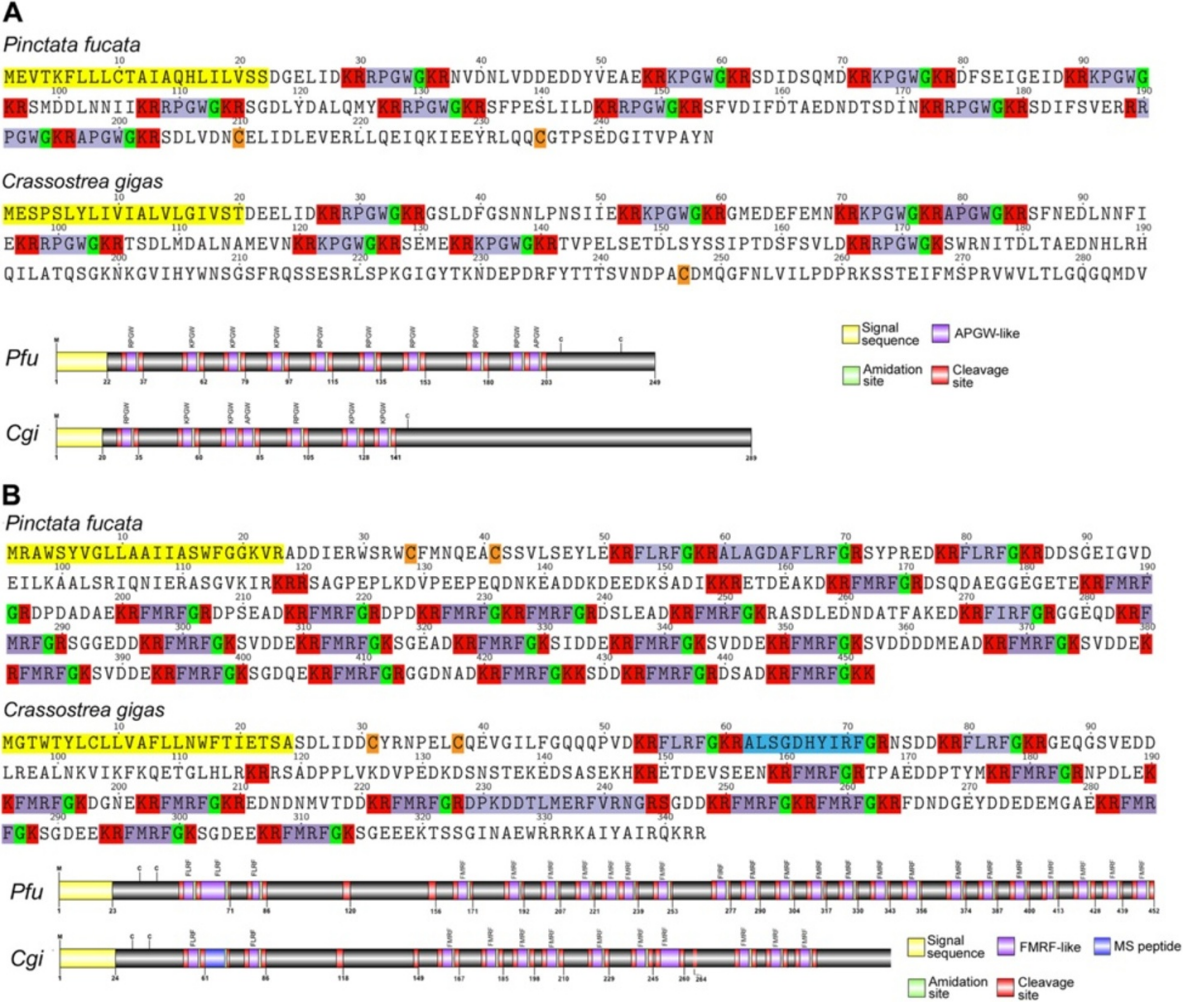
**Identification and characterization of** ***Pinctata fucata*** **and** ***Crassostrea gigas*** **APGW and FMRF precursors. (A)** Amino acid sequence of APGW precursors and schematics showing organization of precursors. **(B)** Amino acid sequence of FMRF precursors and schematics showing organization of precursors.

Molluscan APGWamide and FMRFamide precursors share a similar configuration, that is, they contain numerous tetrapeptide repeats that can vary slightly besides the presence of a C-terminal dipeptide amidation (i.e. GWa and RFa). APGWa precursors previously described in the aquatic gastropods *Aplysia californica* [17] *, Lymnaea stagnalis* [18], *Lottia gigantea* [12] contain primarily APGWa whose putative function has been reviewed by Koene 2010 [19]. The related GWa, TPGWa, KPGWa and RPGWa peptides have been identified in the cuttlefish *Sepia officinalis* [20, 21] and the blue mussel *Mytilus edulis* [22, 23], through HPLC and LC-ESI-MS/MS. Those studies suggest that these related variants might play a significant role in cephalopod and bivalve reproduction. We found that the Pf-APGW precursor is predicted to be cleaved at several dibasic sites, to release six RPGWa peptides (513.3 Da), three KPGWa peptides (485.3 Da) and one APGWa (470.24 Da) (Figure 2A and Additional file 2). Unfortunately no KPGW or RPGW was identified in MS analysis. The Cg-APGW precursor encodes fewer repeats yet has a longer precursor (285 residues). When compared to other APGWa precursors of mollusc, it appears that gastropods more frequently contain the APGWa peptide rather than the TPGWa, RPGWa or KPGWa of bivalves (*P. fucata, C. gigas and M. edulis* [22]). The notable substitution of alanine (A) with threonine (T), arginine (R) or lysine (K) at position 1 in bivalves creates a peptide that is less hydrophobic [22], perhaps imparting a necessary change for it to be bioactive in oysters, mussels and cephalopods.

RFamides, which feature a C-terminal sequence –RFa, constitute one of the largest families of neuropeptides and include the FMRFa and FxRIamide [24–27]. The FMRFa peptides have been described from numerous molluscs, where they have been shown to have a diversity of roles as neurotransmitters, neuromodulators, and neurohormones [28]. The *Pf-FMRF* gene encodes a precursor of 452 residues, including a 23-residue signal peptide, two FLRFa (580.36 Da), twenty FMRFa (598.31 Da), a FIRFa (580.36 Da) and a hydrophobic ALAGDAFLRFa (1078.6 Da) (Figure 2B and Additional file 2). The Cg-FMRF precursor also contains two FLRFa, although fewer FMRFa (ten) and the decapeptide, ALSGDHYIRFa was subsequently identified by mass spectrometry from the visceral ganglia. Interestingly, FMRF precursors exhibit high retention in conservation of the FMRFa with other molluscs but not FLRFa. This may be due to an evolutionary split from a common ancestral precursor [29] and could be unique to bivalves.

Quite similar to the FMRFa is FxRIamide (Additional file 1). This peptide is commonly found in lophotochozoans [30, 31], and has been also called S-Iamide peptide due to its common structure, -SSFVRIamide after it was first reported (LSSFVRIamide) in the prosobranch mollusc *Fusinus ferrugineus* [32]. Since then, partial precursors and fragments of heptapeptides all with the structural amino acid arrangement of xSSFxRI have been reported in the annelids, *Platynereis demurilii*, *Pomatoceros lamarckii*, *Capitella capitata*, and molluscs *Aplysia* and *Lottia* [31]. Here we now also report the identification of full-length precursors of FxRIamide for both oysters, *P. fucata* and *C. gigas*. The *Pf*-*FxRIa* gene encodes leader 22 residue signal peptide as well as 11 unique heptapeptides all with the structural amino acid arrangement of xSSFxRI. Of note, in addition there are two longer peptides IPSSAFMRIa and PSRLGQSSFVRIa. In *C. gigas*, the *Cg-FxRIa* gene has in addition to leader signal peptide, 14 unique FxRI peptides. The unique feature of these FxRI homologs is that they generally are missing the upstream amino acid sequence SS. Only one peptide, verified by MS analysis conformed to the xSSFxRI convention, IQQSSFIRI. Overall the significance of these peptides in molluscs and in annelids is relatively unknown. However, it has been suggested that the peptides may be involved in the regulation of gut motility of the animal [30].

### Gonadotropin-Releasing Hormone and Egg-Laying Hormone

**Figure 3:**
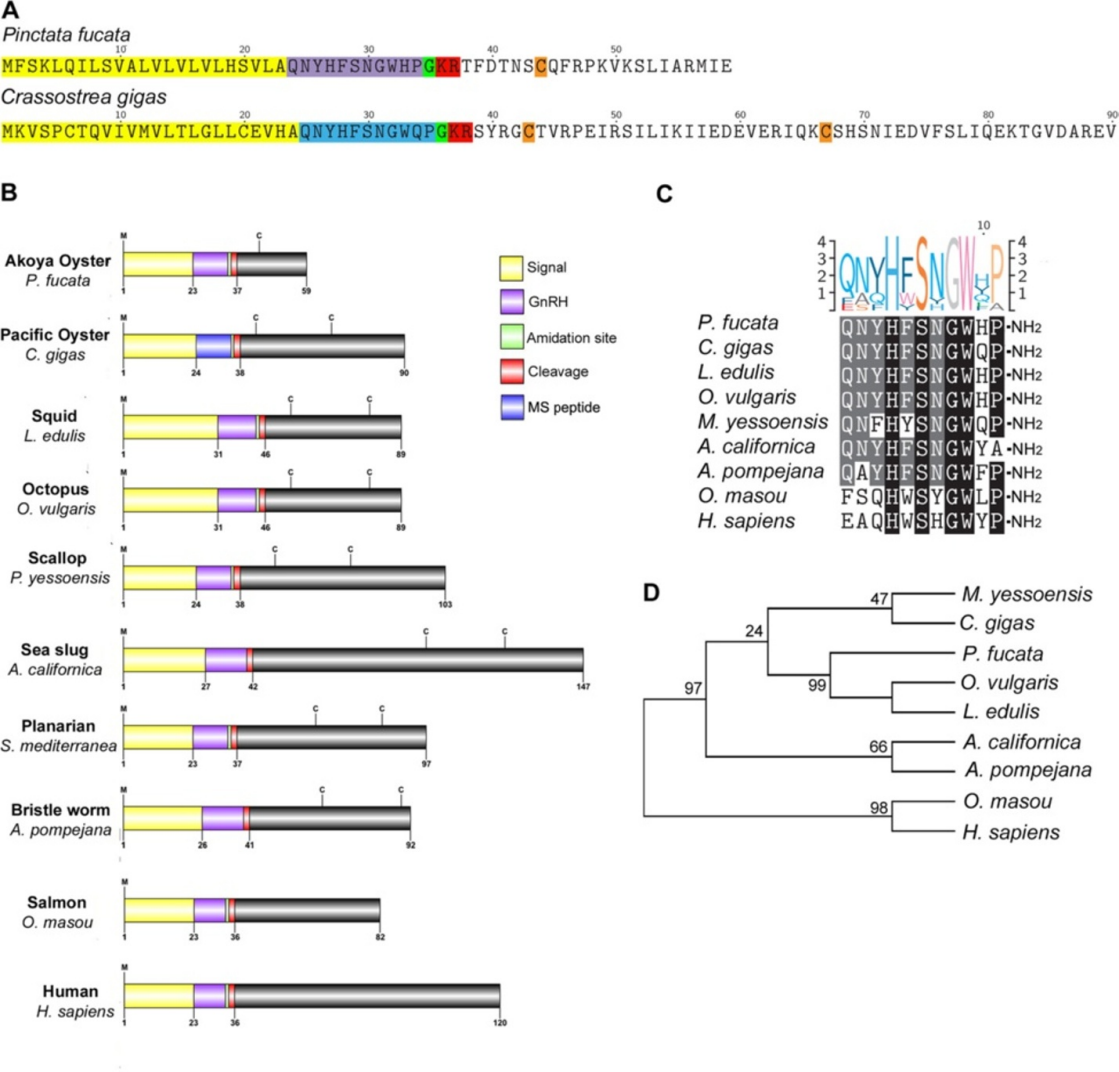
**Identification and characterization of** ***Pinctata fucata*** **and** ***Crassostrea gigas*** **GnRH-like peptides. (A)** Amino acid sequence of GnRH precursors. **(B)** Schematics showing organization of precursors within molluscs and other invertebrates and vertebrates. **(C)** Comparative sequence alignment of GnRH peptide and **(D)** phylogenetic analysis. Sequence logo is shown above alignments.

In oysters, synchronized gonad maturation and spawning is thought to occur via environmental cues, including tides and pheromonal cues released by conspecifics that initiate endogenous cues [33]. The GnRH and ELH peptides have been most well studied in relation to endogenous reproduction hormones. Although much is known about the role of GnRH in vertebrate reproduction [34], we are only just beginning to explore its function in molluscs. In molluscs, a GnRH was first identified in the *Octopus vulgaris* [35]. Later identified also in the sea slug *Aplysia californica,* it was determined that although administration of synthetic GnRH could stimulate behaviours such as inhibition of feeding, no effect on ovotestis, reproductive tract, egg-laying or penile eversion was observed [36]. However, in the scallop *Patinopecten yessoensis*, mammalian GnRH can stimulate spermatogonial proliferation [37]. Two GnRH-related peptides were later found by mass spectrometry of the *C. gigas* visceral ganglia, and gene expression analysis demonstrated a correlation with reproduction and nutritional status [38]. The *Pf-GnRH* and gene identified in the present study encodes for a precursor of 59 amino acids, and includes the conserved 11 residue GnRH-like peptide and C-terminal GnRH-associated peptide (GAP) (Figure 3A). The GnRH-like peptide of *P. fucata* and the previously reported *C. gigas* GnRH differ at only residue 10 (ie. His for *P. fucata* and Gln for *C. gigas*). The general organization of the GnRH precursor has been conserved throughout evolution and with the exclusion of chicken, tree shrew, fish and primates, most species of vertebrate possess one or two cysteines within the GAP (Figure 3B). In comparison with other GnRH peptides, most similarity is found within the GnRH peptide (Figure 3C), and based on phylogenetic analysis of GnRH precursors (Figure 3D) the Pf-GnRH clusters most closely with the cephalopod *Octopus vulgaris*, rather than the bivalves *C. gigas* and *Patinopectin yessoensis*.

**Figure 4:**
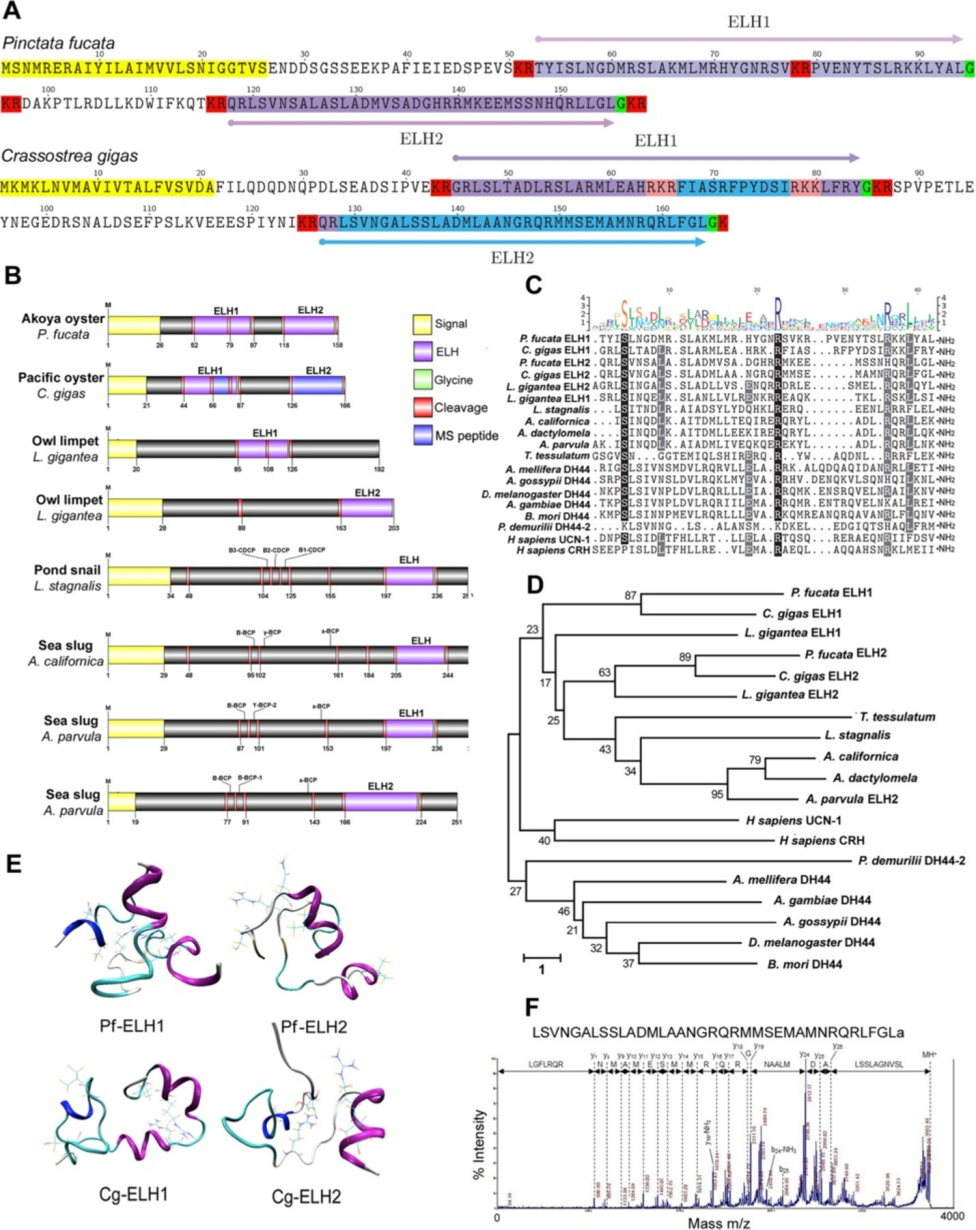
**Identification and characterization of** ***P. fucata*** **and** ***C. gigas*** **ELH-like peptides. (A)** Amino acid sequences of *P. fucata* and *C.gigas* ELH precursors. **(B)** Schematics showing ELH precursors identified in molluscs. **(C)** Comparative sequence alignment and **(D)** Phylogenetic analysis of bioactive ELH/DH44/CRH region between species. Sequence logo is shown above alignments. **(E)** Protein models of oyster ELH1 and ELH2 assembled from molecular dynamics simulation. Secondary structures: blue, helix; purple, alpha-helix; cyan, turn; white, random coil; dark yellow, isolated beta bridge. **(F)** Off-line nLC-MALDI tandem MS analysis of *Crassotrea gigas* cerebral ganglia. MS/MS spectrum of the egg-laying hormone Cg-ELH2 m/z 3939. Immonium, a-, b- and y-ions detected are marked.

The egg-laying hormone has received significant attention due to the nature of its function in eliciting egg laying in *A. californica,* from which it was first discovered [39]. Its gene structure is typical of many molluscan neuropeptides in that it is derived from a precursor that gets processed by processing enzymes, such as prohormone convertase [40]. The *Pf-ELH* gene (pfu_aug1.0_2069.1_29971) primary sequence has been described previously [16] where it encodes a 158 amino acid preprohormone consisting of a 26-residue signal peptide and two ELH-like domains (Figure 4A). Herein, we show that the Pf-ELH1 consists of a 42 amino acid basic peptide (5016.9 Da) and Pf-ELH2 consists of a 38 residue ELH-like acidic peptide (4189.11 Da), both of which are predicted to be cleaved and amidated from a common precursor at S_26_. The Cg-ELH was first described by Veenstra [12], revealing a precursor slightly larger (166 amino acids) than the Pf-ELH, but similarly contains two ELH-like peptides (Pf-ELH1 has 40 residues and Pf-ELH2 has 38 residues).

Schematic representations show that only oysters contain ELH peptides on the same precursor (Figure 4B) and only the *Aplysia* and *Lymnaea* ELH precursors have other known bioactive peptides [such as bag cell peptides (BCPs), caudodorsal cell peptides (CDCPs)]. Multiple sequence comparison and phylogenetic analysis of known ELH peptides shows that oyster ELH1 is most closely related to oyster ELH2 peptides (Figure 4C,D). Most conservation of ELH between molluscan species is located at the N- and C-termini, probably as these are critical for receptor interaction. In support of this, previous structure and activity studies have used synthetic ELH analog variants to investigate egg-laying induction [39]. In that study, removal of the N-terminal amino acid or extension of the C-terminus by one residue (Gly_37_) caused loss of egg-laying activity. Further analysis revealed that ELH is more similar within the *Aplysia* family; where *A. dactylomela* ELH differs from *A. californica* (and *A. brasiliana*) ELH at only four positions [41]. We further compared oyster ELH to the recently identified members of the corticotropin-releasing hormone (CRH) and diuretic hormone 44 [DH44] neuropeptide families reported in other species (Figure 4C). CRH/DH44 have been implicated as being related to mollusc ELH [10], demonstrating sequence similarity within the mature peptides. For example, *Aplysia* ELH compared to DH44, reveals several conserved amino acid positions, including the *Platynereis* DH44, which is highly repetitive (13 and 16 copies) compared to mollusc ELH or insect DH44 counterparts [31]. Human CRH, with 4 identical-semi conserved residues, had the least conservation compared to mollusc ELH, and formed its own sub-branch (Figure 4D). Nevertheless, conservation of these neuropeptides in lophotochozoans further confirms the coevolution of these peptides [10].

No ELH peptide structure model predictions or crystal structures have been reported to date. To help further studies to what may activate an ELH receptor, we undertook predictive structural analysis. Oyster ELH structure molecular models for Pf-ELH1, Pf-ELH2, Cg-ELH1 and Cg-ELH2 predict that ELH1 contains a mixture of helix, α-helix, turn, beta-strand and random coil (Figure 4E). It appears that the highly conserved S_4_ and N_6_ regions exhibit more of a random-unordered structural character, while L_9_ is located within a helix region (i.e., α-helix in Pf-ELH2, 3-10 helix in Cg-ELH1 and Cg-ELH2, respectively). For Cg-ELH2, R_27_ is located within random coil regions, and is adjacent to an α-helix region in Cg-ELH1. Meanwhile, in Pf-ELH1 both R_36_ to L_39_ are within a C-terminal α-helix, which is the same in all ELH models excluding Pf-ELH2. The potential energy as a function of time during this simulation and the backbone root-mean square distance (RMSD) relative to this structure during the course of the same simulation is shown in Additional file 6 (A-D). The representative structure of Pf-ELH1 and Cg-ELH1 occurred at 197.540 ns and 77.028 ns into the MD simulation, respectively. The representative structure of Pf-ELH2 and Cg-ELH2 resolved at 239.778 ns and 100.761 ns into the MD simulation, respectively. Subsequent analysis of the secondary structure of Pf-ELH1 based on circular dichroism (CD) spectroscopy found that the peptide was predominantly beta-strand (36%; peaking at 180-190, and 200-210 nm respectively) (Additional file 6). In addition, the spectrum (particularly the trough from 210-230 nm) indicates a consistent α-helical structure with minor notable random coils throughout (17%; trough 190-200 nm).

Our LC-MS/MS analysis of *C. gigas* neural tissue identified an internal region of Pf-ELH1 (FIASRFPYDSI) and Pf-ELH2 as well as a 36 amino acid form of Cg-ELH2 of *m/z* 3939 (Figure 4F*)*. Whether this form is the biologically active form or just a slightly truncated form remains to be investigated. MS did not detect the 38-residue ELH, probably because its mass exceeds M/Z 4000, which in our analysis was the upper limit of detection.

### Other neuropeptides of interest

**Figure 5:**
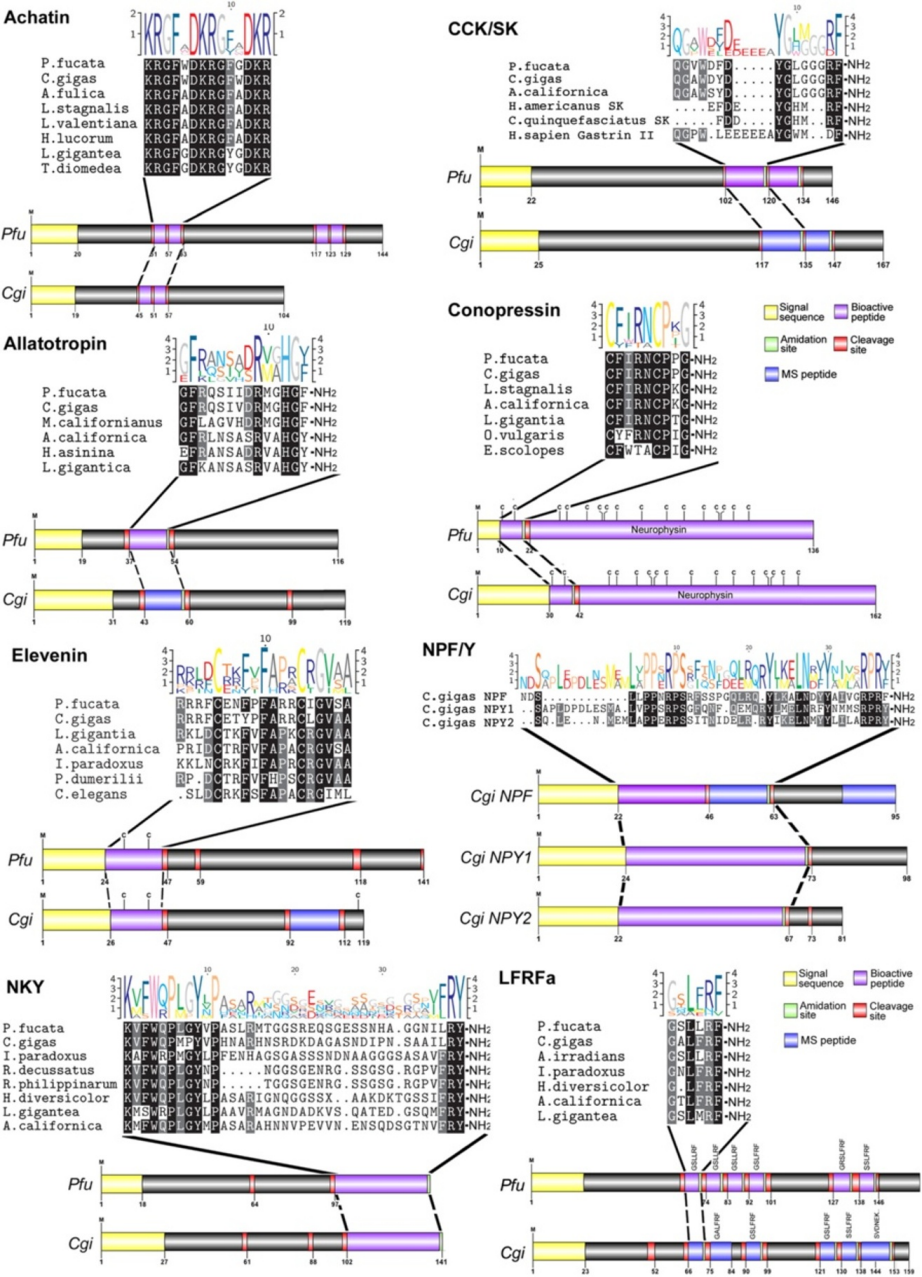
**Identification of** ***Pinctata fucata*** **and** ***Crassostrea gigas*** **neuropeptides.** Comparative sequence alignment and schematic representation of precursors for *P. fucata* (Pfu) and *C. gigas* (Cgi). Full-length precursor sequences were identified in both for achatin, allatotropin, CCK/SK and conopressin, elevenin, NKY, NPF/Y and LFRFa, showing high amino acid identity within bioactive peptides. A sequence logo is shown above alignments, where the height of each letter is proportional to the observed frequency of the corresponding amino acid in the alignment column.

Full-length neuropeptide precursors were identified in *P. fucata* and *C. gigas* for achatin, allatotropin, cholecystokinin (CCK)/sulfakinin (SK)-like, conopressin, elevenin, NKY, NPF/Y and LFRFa (Figure 5). Multiple sequence alignment between the oyster putative neuropeptide precursors with previously identified homolog sequences in mollusc confirms high identity within the bioactive peptide sequences and variability outside these regions. Bioactivity of the intervening sequences has not previously been described.

#### Achatin

The neuropeptide, achatin, was first identified in giant African snail ganglia [42] and within that animal it has both suppressing and enhancing actions on the effects of various neurotransmitters [43]. Similar to other known molluscan achatin precursors, those of *Pf-achatin* and *Cg-achatin* may be processed to release GFWD and GFGD acidic peptides.

#### Allatotropin

The allatotropin peptide stimulates synthesis of juvenile hormones in insects [44], where it has been most well studied. *Pf-allatotropin* and *Cg-allatotropin* encode for allatotropin precursors that can be cleaved to produce a 14 amino acid allatotropin peptide, GFRQSIIDRMGHGFa for *P. fucata* and GFRQSIVDRMGHGFa for *C. gigas* which was confirmed by mass spectroscopy [MS] analysis. While allatotropin is highly conserved between these oysters, there is only limited similarity with other known allatotropins besides the N- and C-terminal regions.

#### CCK/SK

The molluscan oyster homolog genes for CCK/SK encode for peptides of Pf-p-QGVWDFDYGLGGGRFa (1655.79 Da) and Cg-p-QGAWDYDYGLGGGRFamide (1643.74 Da) that share some similarity with sulfakinin and gastrin. An additional co-peptide of Pf-SFGDYSLGGGRFamide and Cg-FDYNFGGGRWamide is also present directly following the CCK/SK peptide. Both *C. gigas* peptides were confirmed by MS analysis (Additional file 3). CCK is structurally and functionally related to the gastrin hormone, both of which regulate digestion and feeding in invertebrates [45].

#### Conopressin

The *Pf-conopressin* and *Cg-conopressin*, encoded a single vasopressin-related conopressin precursor. Immediately following the signal peptide cleavage site is the highly conserved N-terminal conopressin peptide, predicted to be CFIRNCPPG-NH_2_, while the adjoining C-terminal neurophysin peptide contains 14 cysteine residues and is followed by an uncleaved copeptin-homologous domain. Bivalve conopressin differs from gastropod conopressin at the most polymorphic position in the peptide: position 8. In the genetically highly polymorphic *C. gigas,* nucleotide variability even occurs at this position (A/C) changing Q to P. This polymorphic change could be due to a SNP within a population. The conjoined neurophysin-like peptide, although quite divergent, shows spatial conservation of cysteine residues throughout all molluscs as well as in vertebrates and even the NG peptide-associated neurophysins first identified in the sea urchin, *S. purpuratus* [46]. In *Lymnaea*, conopressin controls sexual behavior [47].

#### Elevenin

*Pf-elevenin* and *Cg-elevenin* genes encode an active elevenin peptide that is cleaved from the precursor, KPRRRFCENFPFARRCIGVSA and RRRFGETYPFARRCLGVAA, respectively. LC-MS/MS analysis of *C. gigas* neural tissue identified the SPVSLLEQILNNRRRFGL peptide (Additional file 2), indicating that this is probably a bioactive peptide released from the precursor. Elevenin-like precursors have been described in *Lottia gigantea* [12] and *Aplysia* [48], while a similar precursor from the pygmy squid *Idiosepius* and nematode *Caenhorabditis elegans* are present within the NCBI Genbank database. Comparison of the oyster elevenin precursors with other molluscs and worms shows little overall conservation, even within the elevenin mature peptide, however, there is conservation of cysteine residues at positions 5 and 14.

#### NKY, NPY/F family

Full-length neuropeptide precursors were identified in *P. fucata* and *C. gigas* for NKY and NPY/F. Multiple sequence alignment between the oyster putative neuropeptide precursors with previously identified homolog sequences in mollusc confirms high identity within the bioactive peptide sequences and variability outside these regions. The oyster neuropeptide, neuropeptide KY (NKY) was predicted from precursor cleavage based upon the presence of an N-terminal lysine residue and C-terminal tyrosine residue [12]. The Pf-NKY and Cg-NKY precursors are likely cleaved to release a bioactive peptide of 38 residues with a molecular weight of 4150.64 Da and 4337.84 Da, respectively. Conservation with other NKY exists primarily within the N- and C-terminal regions, while there is limited amino acid identity in the middle region of the precursor. Oyster genome encodes at least three NPY/Fs. Though C-terminal RxRFamide peptides were initially considered as the protostome forms, oysters actually express both a NPF and two NPY peptides. Organization of the oyster genes is similar to those reported in different animal taxa with exons coding for similar domains of the precursor and an intron splicing within the codon of the arginine residue of the C-terminal RF/Yamide sequence [49, 50]. This argues for an early origin of the NPY/F family. In contrast to annelids, oyster genes are however not in tandem position as they are situated on different scaffolds.

#### LFRFamide

The oyster LFRFamide (*Pf-LFRFa* and *Cg-LFRFa*) gene encodes for multiple basic GXLL/FRFa peptides while the LFRYamide (*Pf-LFYa* and *Cg-LFYa*) gene encodes single variant FRF/FRW peptides along with a well conserved associated double disulphide-bridged peptide. While there was no confirmation of *C. gigas* LFRYa peptides by LC-MS/MS, several –LFRFa peptides were. Functionally LFRFa peptides have known to inhibit electrical activity of neuroendocrine cells that control either growth and metabolism or reproduction in *L. stagnalis* [25]. However, in the cephalopod *Sepia officinalis*, LFRFa activity is predominantly targeted to the rectum, where it increases the frequency, tonus and amplitude of rectal contractions [51]. More recently in *C. gigas* where the receptor for this family of peptides had been characterized it has been suggested that signalling of LFRFamide peptides through its specific receptor might play a role in the coordination of nutrition, energy storage and metabolism [52]. In this paper we thus showed the molecular existence of these different peptides by MS/MS in *C gigas* and confirm further via PSI-BLAST molecular analysis that LFRFa would represent functional orthologs of short neuropeptide F from insects [52].

Full and partial-length *P. fucata* and *C. gigas* genes were also identified that encoded peptides with identity to allatostatin C (SHIRCLVNVIACY), buccalins (11 variants), CCAP (VFCNGFFGCSNamide, and LFCNTGGCFamide [although these were not verified by MS analysis]) cerebrin/PDF-like (NLGTVDSLYNLPDLLYRamide), FFamide/SIF-like peptides (GMNPNMNSLFFamide) similar in structure to the FFamides found in *Lymnaea* [53], FCAP, GGNamide (SKCKGPWANHMCFGGNamide), LASGLVamide (MMDPLASGLVa), LFRYa (SIKIPFRFa), luqin (APQWRPQGRFamide and VCVESNVPGLFKCY), two myomodulin variants (GMPMLRLamide, PFKMLRLamide GGLSMLRL, GLQMLRLamide, and AMPMLRLamide) and (xG/KFFRIamide), Pedal peptide, PKYMDT, sCAP (small Cardio Active Peptide)/pyrokinin-like peptides (APKYFYFPRMamide; SAFYFPRMamide); tachykinins (FGFAPMRamide, 824.01 Da; FRFTALRamide 909.09 Da), and the NdWFamide (Additional file 1). We also present for the first time the expression of opioid-like neuropeptides, considered to represent the protostome counterparts of enkephalins [2] (Additional file 1). Novel neuropeptide families displaying any obvious similarity to known peptides were named PFGx8amide, RxIamide and Wx_3_Yamide according to their sequence pattern. These new families may represent mollusc or Lophotrochozoan innovations. We found no neuropeptide related to the gastropod mollusc pleurin, sensorin and enterin, however a neuropeptide PXVFamide consensus sequence corresponding exactly to the active core of the *Mytilus* inhibitory peptides and PXVFamide found in *Aplysis* and *Lottia* was characterized [12]. LC-MS/MS analysis of *C. gigas* neural tissues allowed the characterization of the majority of the predicted neuropeptides with the exception of those excluded by the mass sieve applied (600 Da -4000 Da) or those harboring an intrapeptide disulfide bridge which, in absence of a reduction/alkylation step, usually do not generate interpretable mass fragments (allatostatin C, conopressin, GGNamide). Some other peptides were virtually not detected, as is the case for LASGLVamide family of neuropeptides or Luqin. We cannot rule out the possibility that the extraction procedure was not adequate or that the mature peptides are expressed at very low levels in the adult nervous system. The findings that the genes encoding these peptides (OYG_10000034, OYG_10021332) are highly expressed in pediveliger larvae highlight that there is a more specific role for these peptides during larval development (see OysterDB; http://oysterdb.cn/). The peptidomic approach confirmed the expression and the actual processing of virtually all predicted peptides. Nevertheless, some intriguing features were uncovered; these include the presence of few glycine C-terminally extended peptides together with their amidated forms (FFamide, GnRH, *Mytilus* inhibitory peptide [PXFVamide], Myomodulin and sCAP) as well as some extended peptides with internal processing sites (FFamide, myomodulins, pedal peptide 3, PKYMDT and NPYamide). In addition, few unpredicted peptides were also characterized (LRNFVa and NPF). Whether these peptides represent biologically active moieties or simply incomplete processed forms remain to be further investigated.

Sequence alignments of oyster neuropeptide precursors with corresponding molluscan precursors show conservation only within the putative bioactive peptides; further investigation was needed to fully comprehend their diversity and evolution in molluscs (for review, see [12]). Therefore, we used similarity-based clustering and sensitive similarity searches. Clustering recovered all known molluscan neuropeptide families (Additional file 7), several of which are unique with no connections to other families (e.g. neuropeptide precursors cerebrin, PKYMDT, bursicon alpha, elevenin, allatostatin C, sCAP/Pyrokinin, NKY, GPA2 and GnRH). However, 17 of the 42 families were strongly connected to form one large central cluster; although in the central cluster some sequences were only indirectly connected via a network of transitive BLAST connections (e.g. achatin, FFamide, luqin, CCAP and PFGx8amide). Not surprisingly, the core of the central cluster represented and contained neuropeptides with abundant repetitive peptides that give rise to short tetra to dodeca amidated (e.g. APGW, LASGL, FMRF, LFRF, mytilus inhibitory peptide, FFamide, PFGx8amide, myomodulin), and nonamidated neuropeptides (e.g., pedal peptide). This is similar to the observations made in a few recent cluster-based studies of neuropeptide families encompassing far larger datasets and not restricted to one phyla [2, 31]. Several peripheral groups (e.g., ELH, conopressin, NPF/NPY and CCK/SK) were connected to the core, but not to other derived families. This adheres to the observation that these fringe neuropeptides represent independent divergences from one or more ancestral sequences within the core [2]. The protein family from opioid, Wx3Yamide, achatin-like, bursicon beta, GPB5, tachykinin, LFRYamide, NdWFamide did not mark in the map because i) their similarity to others was higher than 1e^-5^; and ii) they did not form a cluster in the map.

### Glycoproteins

**Figure 6:**
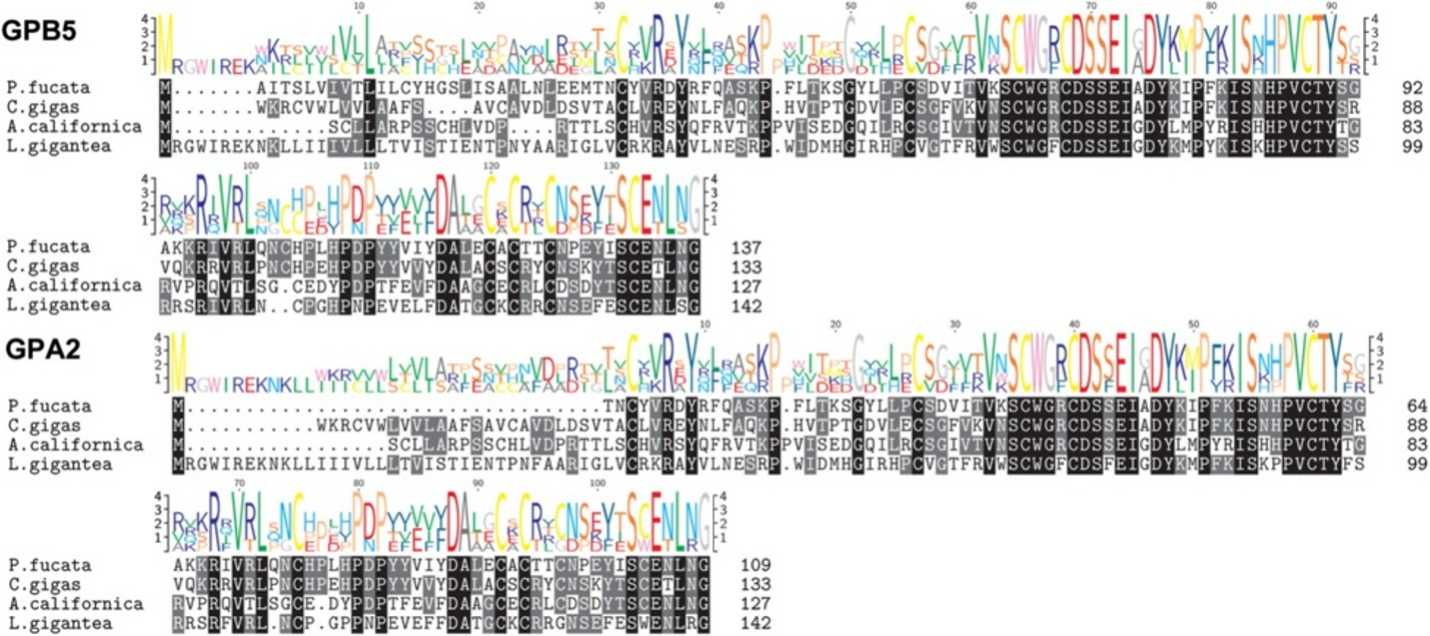
**Identification of GBP5 and GPA2 in oysters.** Comparative sequence alignments of GBP5 and GPA2 precursors for *Pinctada fucata* and *Crassotrea gigas* with the gastropods *Aplysia californica* and *Lottia. gigantea*. Sequence logo is shown above alignments.

A glycoprotein family known as cysteine knot-forming heterodimers consisting of alpha- (GPA) and beta-subunits (GPB) are evolutionarily conserved [54]. In vertebrates the heterodimer is called thyrostimulin, composed of GPB5 and GPA2. Homologs occur in arthropods, nematode, cnidarians, and molluscs implying that this neurohormone system existed prior to the emergence of bilateral metazoans [55]. The GPB5 of the GPA2/GPB5 dimer was identified in *P. fucata* and *C. gigas* that is similar to the GPB5 subunits identified in *Aplysia*, *Lottia* [12] and humans [56] (Figure 6 and Additional file 1). The *Pf-GPB5* and *Cg-GPB5* genes encode precursors of 137 and 133 residues, respectively, and two GPB5 peptides are predicted to be cleaved from these precursors, releasing N-terminal basic GPB5 (8193.32 Da) and a C-terminal acidic GPB5 (4708.33 Da), both of which contain five cysteine residues. GPA2 was also identified from *P. fucata* and *C. gigas* (Figure 6 and Additional file 1). Cg-GPA2 is encoded by a gene in tandem with *Cg-GPB5*, as is the case in most species [57]. There is spatial conservation of cysteine residues amongst molluscan GPB5/GPA2, yet only the oysters retain the KR cleavage site. Although a function for these proteins in molluscs is currently unknown, in insects, studies using the mosquito (*Aedes aegypti*) suggest that GPA2/GPB5 participates in ionic and osmotic balance, since it appears to inhibit natriuresis and promote kaliuresis [55]. Bursicon, another αβ heterodimer member of this cysteine knot family of neurohormones, was characterized in insects for its role in triggering the sclerotization of the cuticle and expansion of the wings during the final phase of metamorphosis [58]. Both α and β bursicon-related precursors are encoded by *C. gigas* genome. Cg-Bursicon α, a 108 amino acid long peptide, shows 53% similarity with *Bombyx mori* bursicon-β subunit, though Cg-Bursicon-β (displays 46% identity with *Carcinus maenas* counterpart. Cg-Bursicon-αβ very likely binds oyster receptor Cg-LGRB with a possible growth/differentiation regulatory role during development and in the cytological changes occurring in the digestive gland [59].

## Conclusions

In this study we described the identification of putative oyster neuropeptides using *in silico* genome and transcriptome database searches. The results clearly demonstrate that neuropeptide genes are conserved in bivalves, however, there are distinct differences with other molluscs. Despite a sessile mode of life and thus less intricate patterns of behavioral events, oysters have obviously retained a repertoire of neuropeptides with a complexity similar to that of other mollusc classes. The number of peptides predicted in our study supports the power of genome mining for neuropeptide gene discovery, and provides a strong foundation for future *in silico* investigations within oysters. Further research is additionally needed to validate peptide predictions through gene expression analysis as well as peptide expression identification using mass spectrometry approaches with other endocrine tissues and at different stages of development and metabolic states. To achieve this, target tissues would include the oysters visceral and cerebral ganglia, gonads and in depth *in vivo* assays of synthetic and recombinant peptides. Function must then be confirmed by bioactivity.

## Methods

### Gene and peptide identification

To identify target sequences, the *Pinctata fucata* (http://marinegenomics.oist.jp/genomes/download?project_id=20) and *Crassostrea gigas* (http://gigadb.org/pacific_oyster) genome [60] and gene coding region (CDS) databases were imported into the CLC Genomics Workbench (v6.0; Finlandsgade, Dk). Previously identified molluscan neuropeptides, neurohormones and precursor processing enzyme sequences were then used to query (tBLASTn and BLASTx) the databases. In parallel, open reading frames retrieved from the databases were translated and screened for the presence of recurrent KK; KR; RK; RR motifs. In many cases, *C. gigas* gene CDS predictions could be supported from transcriptome database analyses. Multiple sequence alignments were created with the Molecular Evolutionary Genetics Analysis (MEGA) software version 5.1 [61]. Derived and actual amino acid sequences were aligned, guided by chain cleavage sites and conserved cysteines, where necessary intron donor/acceptor splice sites were identified using NetGene2 [62]. Signal sequences and cleavage sites were identified by alignments with other mollusc sequences [12, 63, 64] and predicted through SignalP 4.0 [5] and NeuroPred [65]. Sequence presentation and shading of multiple sequence alignments was performed using the LaTEX TEXshade package [66].

### Phylogeny and neuropeptide family clustering

Phylogenetic trees were constructed using full length precursors or individual peptides with MEGA5.1 utilising the neighbor-joining method [67]. Unrooted trees were generated with 1000 bootstrap trials and presented with a cut-off bootstrapping value of 50. For the neuropeptide family classification, we performed a PSI-BLAST with 1 iteration using all the molluscan neuropeptides identified in this study. The sequence-similarity-based clustering approaches were further applied using CLANS [68]. All the neuropeptides sequences with no similarity to other neuropeptides were removed from the final map.

### Precursor schematics and peptide modelling

Schematic diagrams of protein domain structures were prepared using the Domain Graph (DOG, version 2.0) software [69]. Protein secondary structure predictions were made using PredictProtein (http://www.predictprotein.org/), and protein 3D models were built using the Assisted Model Building with Energy Refinement (AMBER) 11 [70]with a modified procedure described elsewhere [71], in which the structures of molecular dynamic simulation were sampled every picosecond for a total of 350 nanoseconds, and a representative structure (i.e. the lowest energy structure) was obtained once the RMSD during a long time period was below ~2 Å. To characterize the secondary structure of *P. fucata* ELH, CD analysis was performed using a 0.1 cm path length cell at 0.2 nm intervals with two scans (178–260 nm) averaged for each at 25°C (Jasco J-715 spectropolarimeter, JASCO, Easton, MD, USA). Lyophilized ELH was slowly dissolved in 10 mM sodium phosphate buffer (pH 6.5), centrifuged at 15,000 × g to remove precipitated protein. It was then concentrated to a volume of 0.5 ml by centrifugation at 7000 × g in a Centricon 3 concentrator (Amicon, Beverly, MA, USA). One more addition of buffer and concentration was performed, after which the protein was diluted with additional sodium phosphate buffer to a final concentration of 0.3 mg**/**ml. Far-UV CD spectra were taken at a protein concentration of 0.1 mg/L and the resultant spectra corrected for the buffer signal. The CD spectrum of *P fucata* ELH was interpreted with the program, Contin using the DICHROWEB site [72–74].

### Animals and extraction of peptides from visceral ganglia and nano-LC purification

All experiments were conducted in accordance within Australian laws, and laws imparted by French counterparts, and thus required no ethics approval for the animals used in the study.

Two-year old adult *C. gigas* purchased from an oyster farm (Normandie, France) were used for peptide identification. Twenty animal equivalents of visceral ganglia were extracted in 0.1% trifluoroacetic acid (TFA) at 4°C and centrifuged for 30 min at 35,000 × g at 4°C. The supernatants were concentrated on Chromafix C_18_ solid phase extraction cartridges (Macherey-Nagel). Samples were evaporated and nano-LC purification performed as described in Bigot *et al.* [38].

### Mass spectrometry analysis

MS analysis were carried out on an AB Sciex 5800 proteomics analyzer equipped with TOF–TOF ion optics and an OptiBeamTM on-axis laser irradiation with 1000 Hz repetition rate. The system was calibrated immediately before analysis with a mixture of des- Arg-Bradykinin, Angiotensin I, Glu1-Fibrinopeptide B, ACTH (18-39), ACTH (7-38) and mass precision was above 50 ppm. A 0.8 μl volume of the HPLC fraction was mixed with 1.6 μl volume of a suspension of CHCA matrix prepared in 50% ACN/0.1% TFA solvent. The mixture was spotted on a stainless steel Opti-TOFTM 384 targets; the droplet was allowed to evaporate before introducing the target into the mass spectrometer. All acquisitions were taken in automatic mode. A laser intensity of 3000 was typically employed for ionizing. MS spectra were acquired in the positive reflector mode by summarizing 1000 single spectra (5 × 200) in the mass range from 600 to 4000 Da. MS/MS spectra were acquired in the positive MS/MS reflector mode by summarizing a maximum of 2500 single spectra (10 × 250) with a laser intensity of 3900. For the tandem MS experiments, the acceleration voltage applied was 1 kV and air was used as the collision gas. Gas pressure medium was selected as settings. The fragmentation pattern was used to determine the sequence of the peptide. Database searching was performed using the Mascot 2.3.02 program (Matrix Science) from the latest version of *C. gigas* transcriptome “GigasDatabase” [60] (including 1,013,570 entries) http://publiccontigbrowser.sigenae.org:9090/Crassostreagigas/index.html and *C. gigas* genome sequence database http://oysterdb.cn/. The variable modifications allowed were as follows: C-terminal amidation, N-terminal pyroglutamate, N-terminal acetylation, methionine oxidation and dioxidation. Mass accuracy was set to 300 ppm and 0.6 Da for MS and MS/MS mode respectively. Mascot data were then transferred to an in-house developed validation software for data filtering according to a significance threshold of Mascot score >20 and the elimination of protein redundancy on the basis of proteins being evidenced by the same set or a subset of peptides. Each peptide sequence was checked manually to confirm or contradict the Mascot assignment. Sequences corresponding to irrelevant identifications were discarded.

## Electronic supplementary material

Additional file 1: **Genes encoding putative full-length or partial-length neuropeptide precursors from the** ***Pinctada fucata*** **and** ***Crassostrea gigas*** **genome, and transcriptome databases for** ***C. gigas.*** (PDF 3 MB)

Additional file 2: **Summary of neuropeptide precursors and cleaved products predicted from** ***Pinctata fucata*** **and** ***Crassostrea gigas***. Blue colored peptides indicate those identified from visceral ganglia by mass spectrometry. Database Accession numbers for sequences used in this study. (XLS 138 KB)

Additional file 3: **Off-line nLC-MALDI tandem MS analysis of** ***C. gigas*** **cerebral ganglia.** MS/MS spectrum of the neuropeptides Cg-buccalin: GLDRYSFYGGLa *m/z* 1246.6, Cg-cerebrin; NLGTVDSLYNLPDLLYRa *m/z* 1965, Cg-FFamide: GMNPNMNSLFFa *m/z* 1270.6. Immonium, a-, b- and y-ions detected are marked. (PDF 103 KB)

Additional file 4: **List of peptides molecularly characterized by nLC-MALDI tandem MS analysis of oyster cerebral ganglia Peptide sequence was validated according to a significance threshold of Mascot probability based score >20 and checked manually to confirm or contradict the Mascot assignment.** Na.a and Ca.a: flanking amino and carboxy amino acids on the precursor. (XLSX 26 KB)

Additional file 5: **Complete list of neuropeptides in a FASTA format.** (TXT 68 KB)

Additional file 6: **Potential energy of** ***Pinctada fucata*** **and** ***Crassostrea gigas*** **ELH1 and ELH2 as a function of time during MD (Figures** 1 **-** 4 **).** Figure 5 shows the results of analysis of the secondary structure of Pf-ELH1 based on CD spectroscopy. (PDF 899 KB)

Additional file 7: **PSI-BLAST cluster map of all the molluscan neuropeptides used in this study.** Nodes are colored based on protein family. Edges represent the BLAST connections of *P* value < 1e^-5^. The identifier of oyster neuropeptides is provided in Figure 1, and all molluscan neuropeptides in Additional file 2. (TIFF 2 MB)

## Abbreviations

LC: Liquid chromatography
MALDI-TOF: Matrix-assisted laser desorption/ionization time-of-flight
MS: Mass spectrometry
RMSD: Root-mean square distance
ELH: Egg-laying hormone
FCAP: Feed circuit activating peptide
GnRH: Gonadotropin-releasing hormone
GPBA: Glycoprotein alpha
GPB5: Glycoprotein beta
NPF: Neuropeptide F
NKY: Neuropeptide KY
NPY: Neuropeptide Y
CCK: Cholecystokinin
SK-like: Sulfakinin.
