# Supplementary figures and images for: Neuropeptides encoded by the genomes of the Akoya pearl oyster Pinctata fucata and Pacific oyster Crassostrea gigas: a bioinformatic and peptidomic survey
Source: BMC Genomics. 2014 Oct 2;15(1):840. doi: 10.1186/1471-2164-15-840 (PMC4200219; doi:10.1186/1471-2164-15-840)

# Buccalin

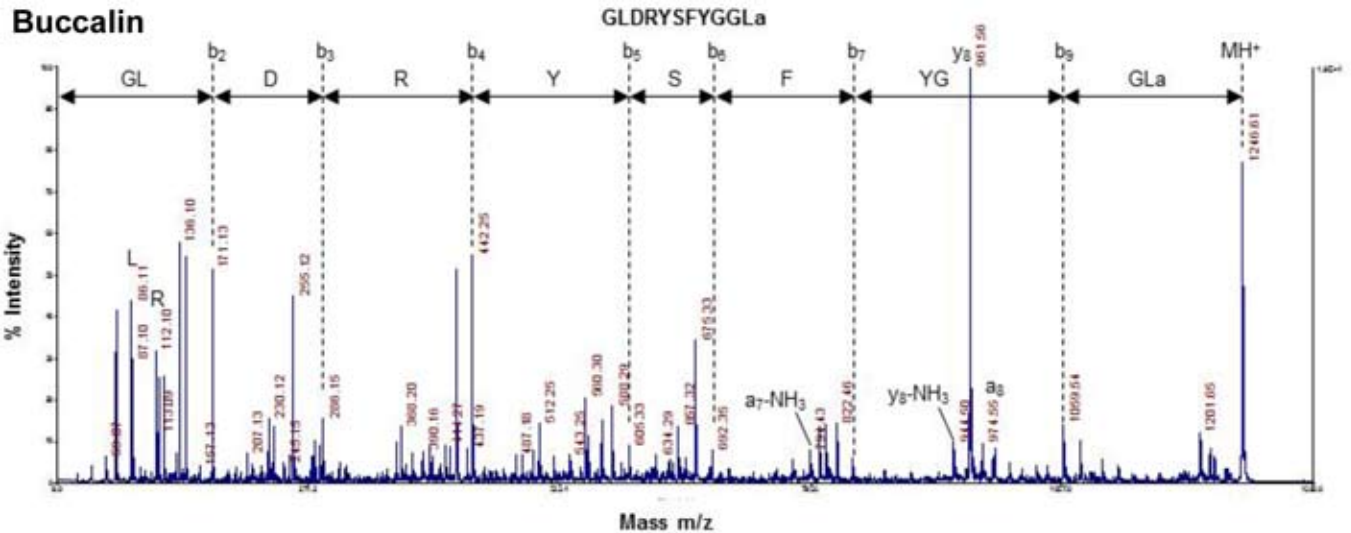

# Cerebrin

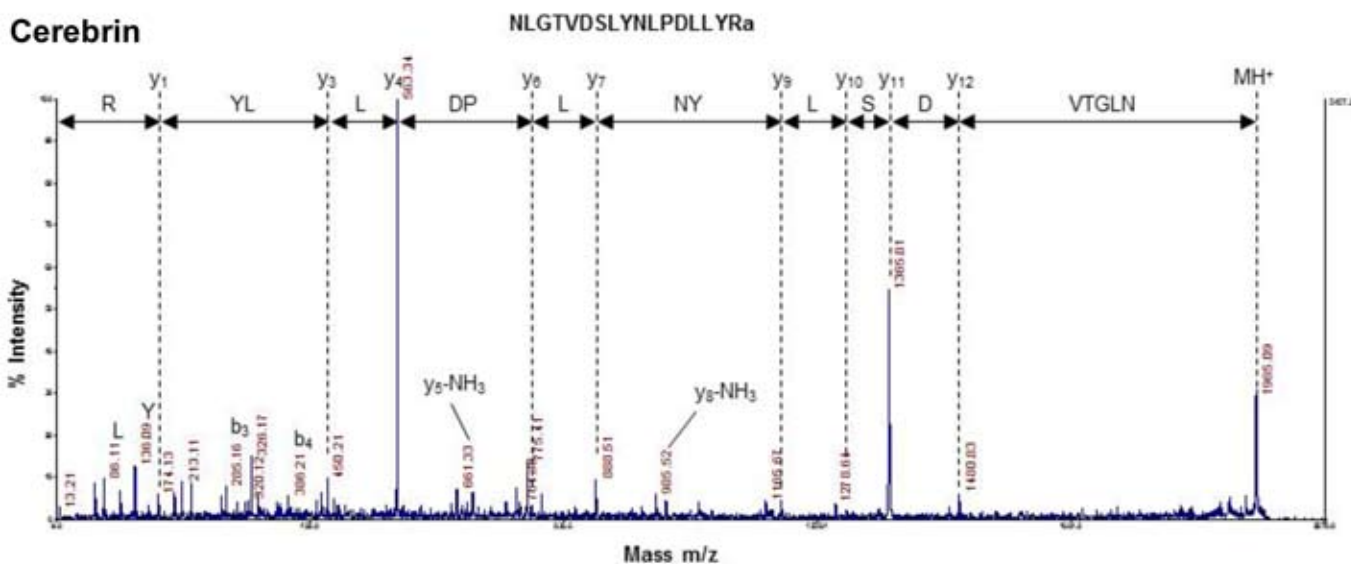

# FFa

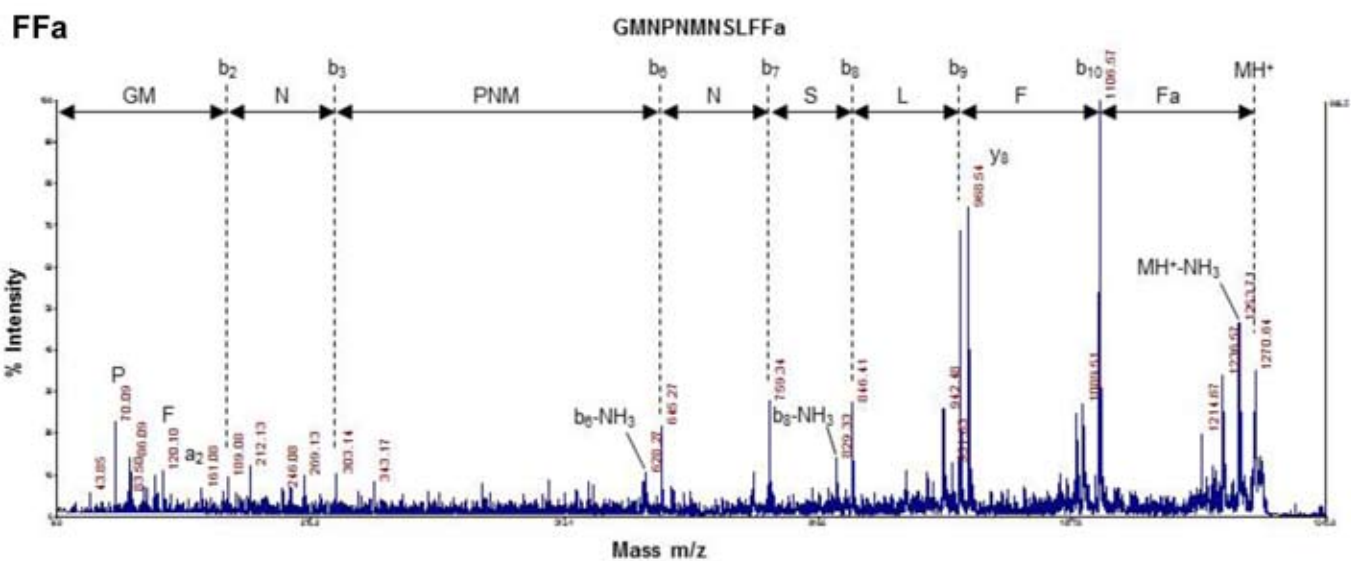

Supplement: Supplementary file 3 — Additional file 3: Off-line nLC-MALDI tandem MS analysis of C. gigas cerebral ganglia. MS/MS spectrum of the neuropeptides Cg-buccalin: GLDRYSFYGGLa m/z 1246.6, Cg-cerebrin; NLGTVDSLYNLPDLLYRa m/z 1965, Cg-FFamide: GMNPNMNSLFFa m/z 1270.6. Immonium, a-, b- and y-ions detected are marked. (PDF 103 KB) [file 12864_2014_6547_MOESM3_ESM.pdf]

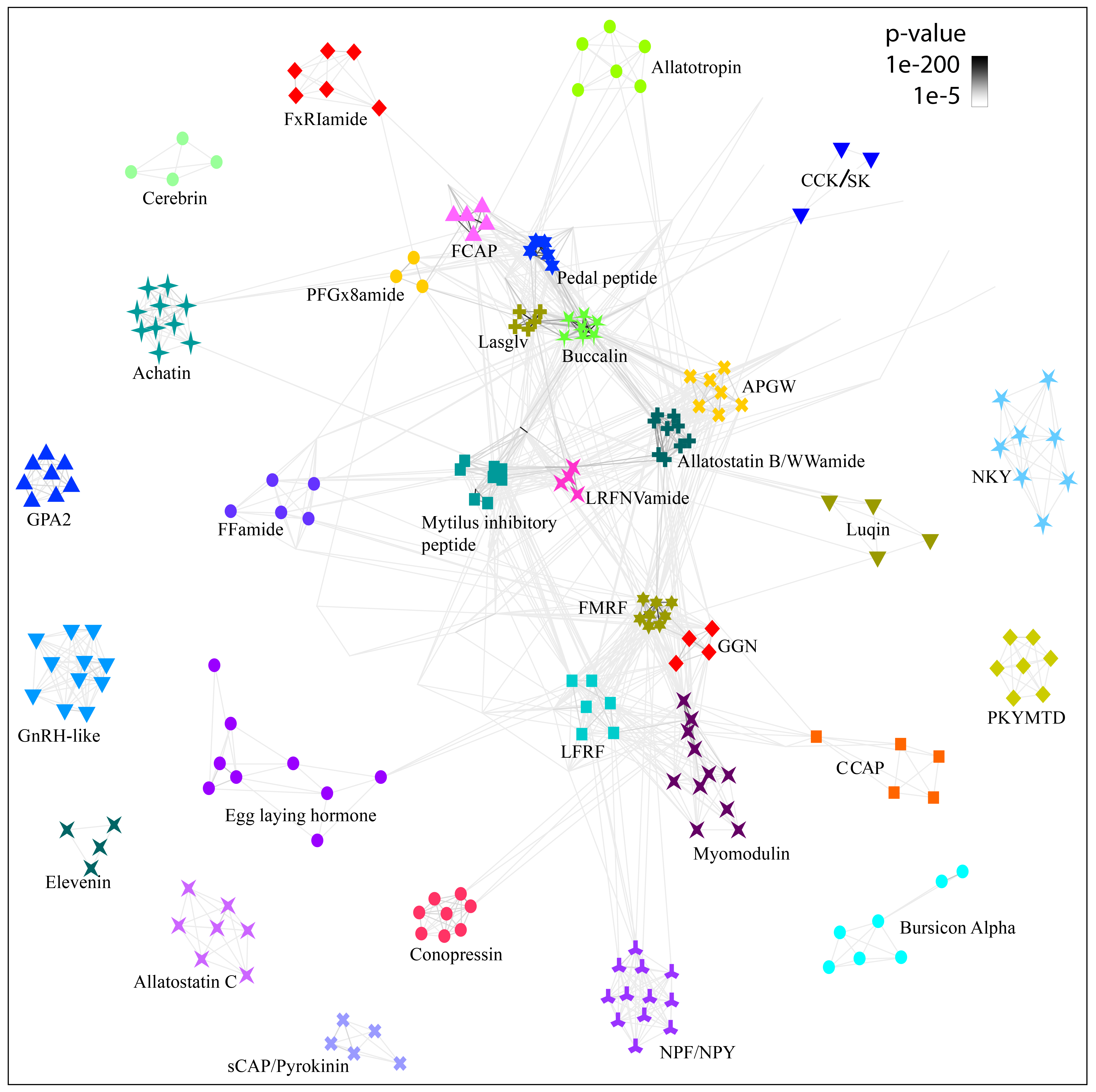

Supplement: Supplementary file 7 — Additional file 7: PSI-BLAST cluster map of all the molluscan neuropeptides used in this study. Nodes are colored based on protein family. Edges represent the BLAST connections of P value < 1e-5. The identifier of oyster neuropeptides is provided in Figure 1, and all molluscan neuropeptides in Additional file 2. (TIFF 2 MB) [file 12864_2014_6547_MOESM7_ESM.tiff]
