# Supplementary material for: Neuropeptides encoded by the genomes of the Akoya pearl oyster Pinctata fucata and Pacific oyster Crassostrea gigas: a bioinformatic and peptidomic survey
Source: BMC Genomics. 2014 Oct 2;15(1):840. doi: 10.1186/1471-2164-15-840 (PMC4200219; doi:10.1186/1471-2164-15-840)
Supplement: Supplementary file 1 — Additional file 1: Genes encoding putative full-length or partial-length neuropeptide precursors from the Pinctada fucata and Crassostrea gigas genome, and transcriptome databases for C. gigas. (PDF 3 MB) [file 12864_2014_6547_MOESM1_ESM.pdf]

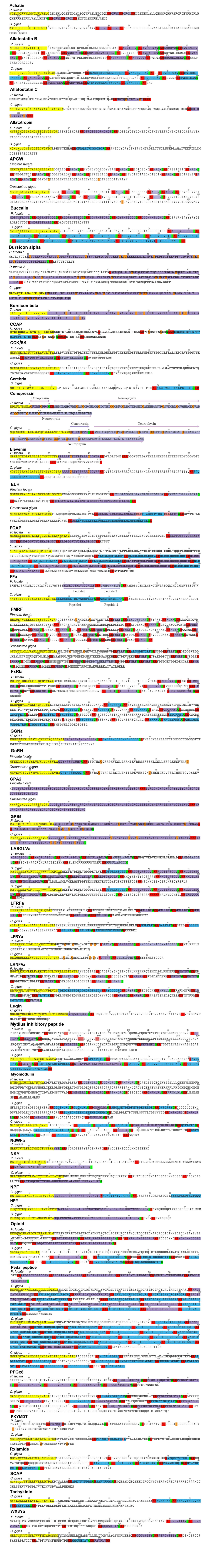
Achatin
Allatostatin B
Allatostatin C
Allatotropin
APGW
Buccalin
Bursicon alpha
Bursicon beta
CCAP
CCK/SK
Cerebrin
Conopressin
Elevenin
ELH
FCAP
FFa
FMRF
FxRIa
GGNa
GnRH
GPA2
GPB5
LASGLVa
LFRFa
LFRYa
LRNFVa
Luqin
Mytilus inhibitory peptide
Myomodulin
NdWFa
NKY
NPF
NPY
Opioid
Pedal peptide
PFGx8
PKYMDT
Rxlamide
SCAP
Tachykinin
WX3Ya
