# Supplementary material for: Neuropeptides encoded by the genomes of the Akoya pearl oyster Pinctata fucata and Pacific oyster Crassostrea gigas: a bioinformatic and peptidomic survey
Source: BMC Genomics. 2014 Oct 2;15(1):840. doi: 10.1186/1471-2164-15-840 (PMC4200219; doi:10.1186/1471-2164-15-840)
Supplement: Supplementary file 6 — Additional file 6: Potential energy of Pinctada fucata and Crassostrea gigas ELH1 and ELH2 as a function of time during MD (Figures 1 - 4 ). Figure 5 shows the results of analysis of the secondary structure of Pf-ELH1 based on CD spectroscopy. (PDF 899 KB) [file 12864_2014_6547_MOESM6_ESM.pdf]

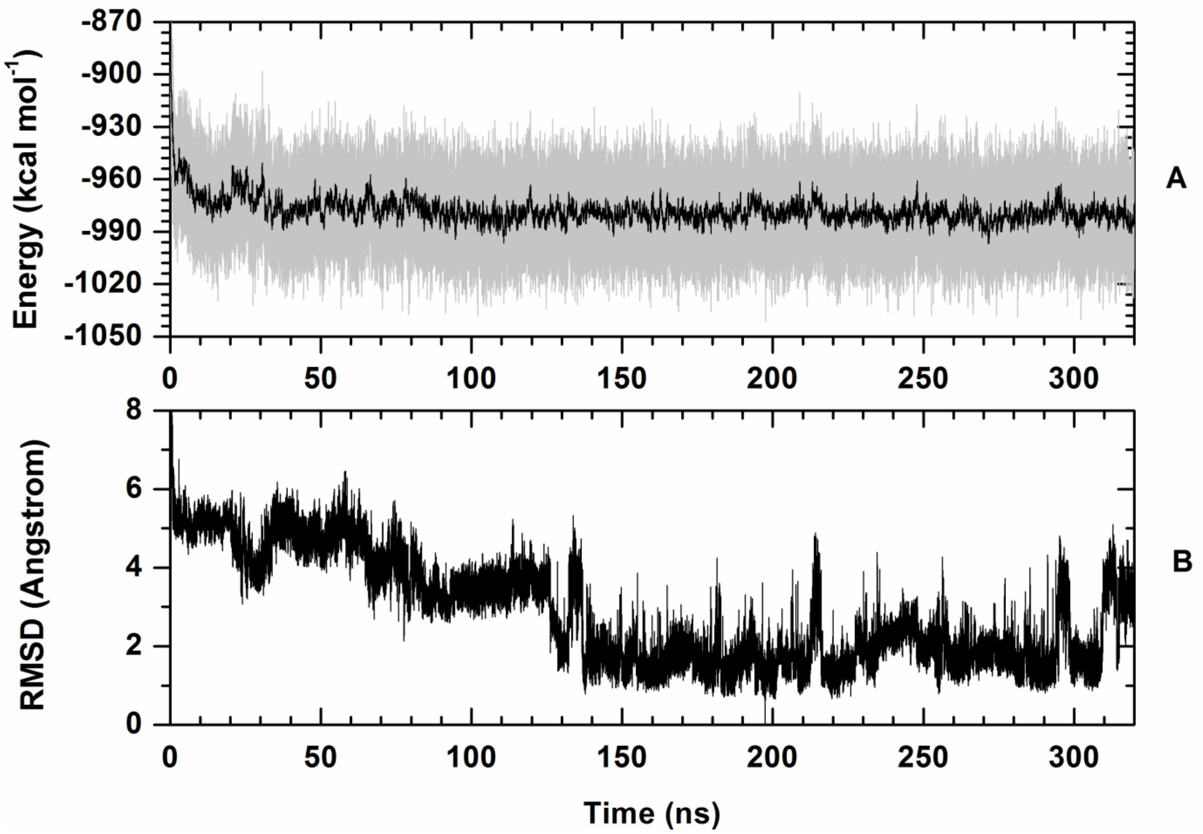

Figure 1 (A) Potential energy of Pfu-ELH1 as a function of time during MD. The solid line is a running average over 100 ps. (B) Backbone rmsd during the same MD, compared to the lowest-energy conformation (the representative structure).

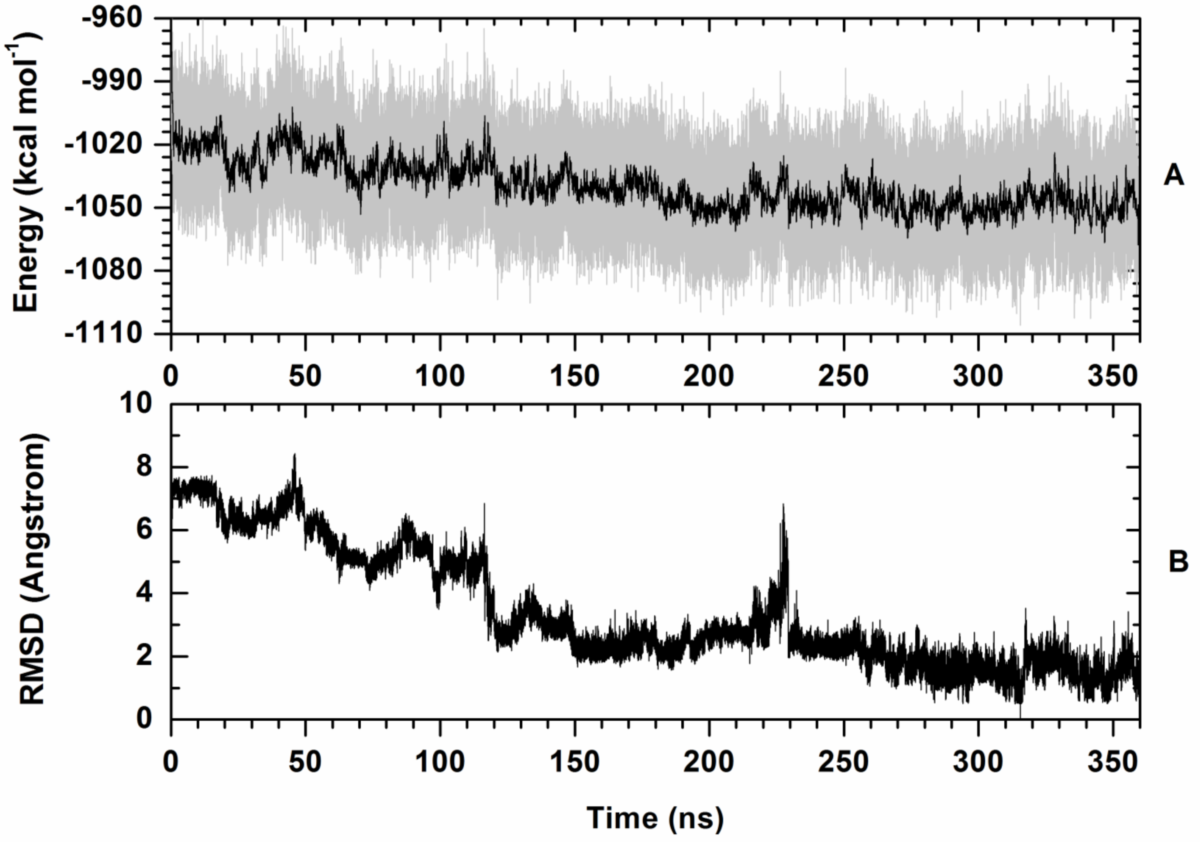

Figure 2 (A) Potential energy of Pfu-ELH2 as a function of time during MD. The solid line is a running average over 100 ps. (B) Backbone rmsd during the same MD, compared to the lowest-energy conformation (the representative structure).

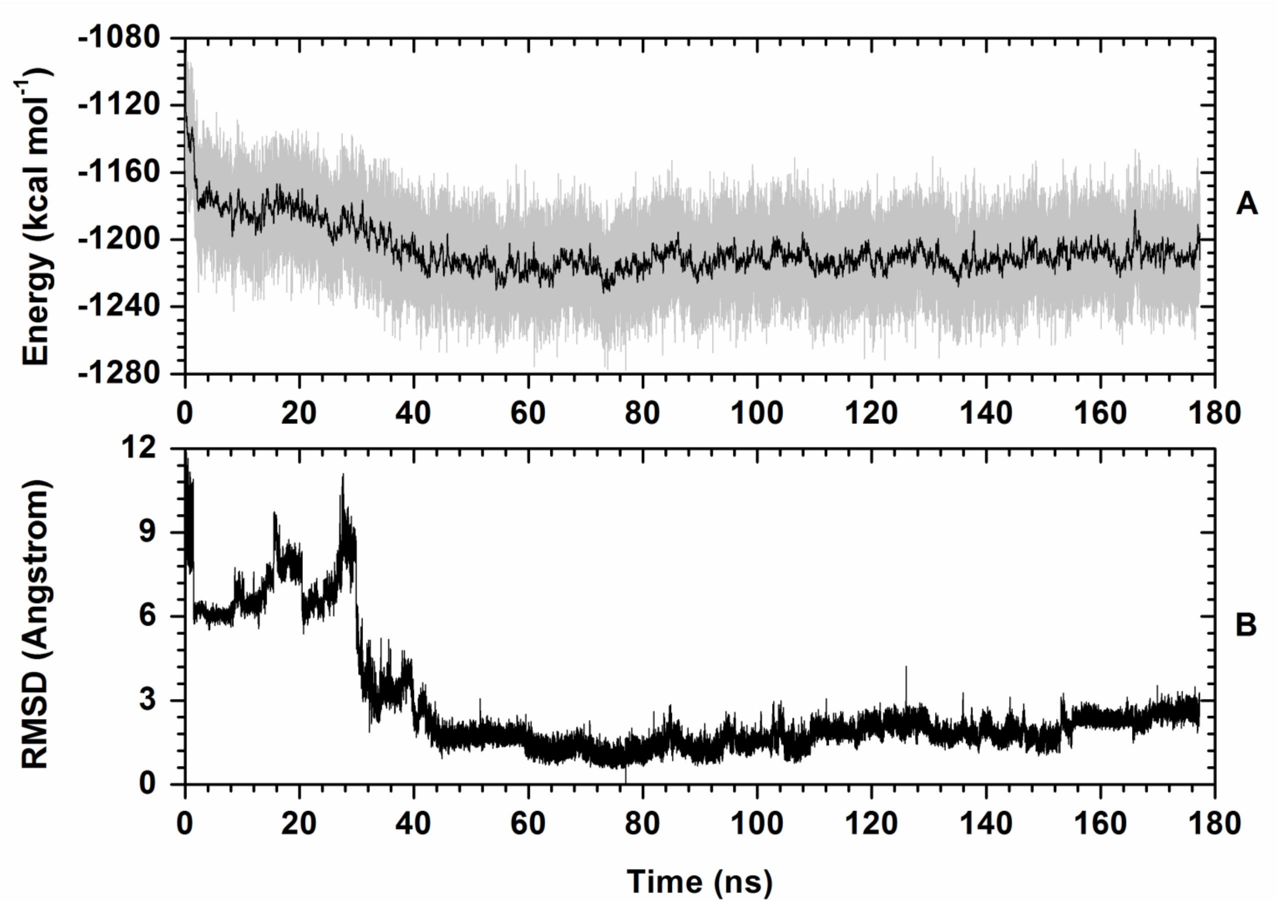

Figure 3 (A) Potential energy of Cgi-ELH1 as a function of time during MD. The solid line is a running average over 100 ps. (B) Backbone rmsd during the same MD, compared to the lowest-energy conformation (the representative structure).

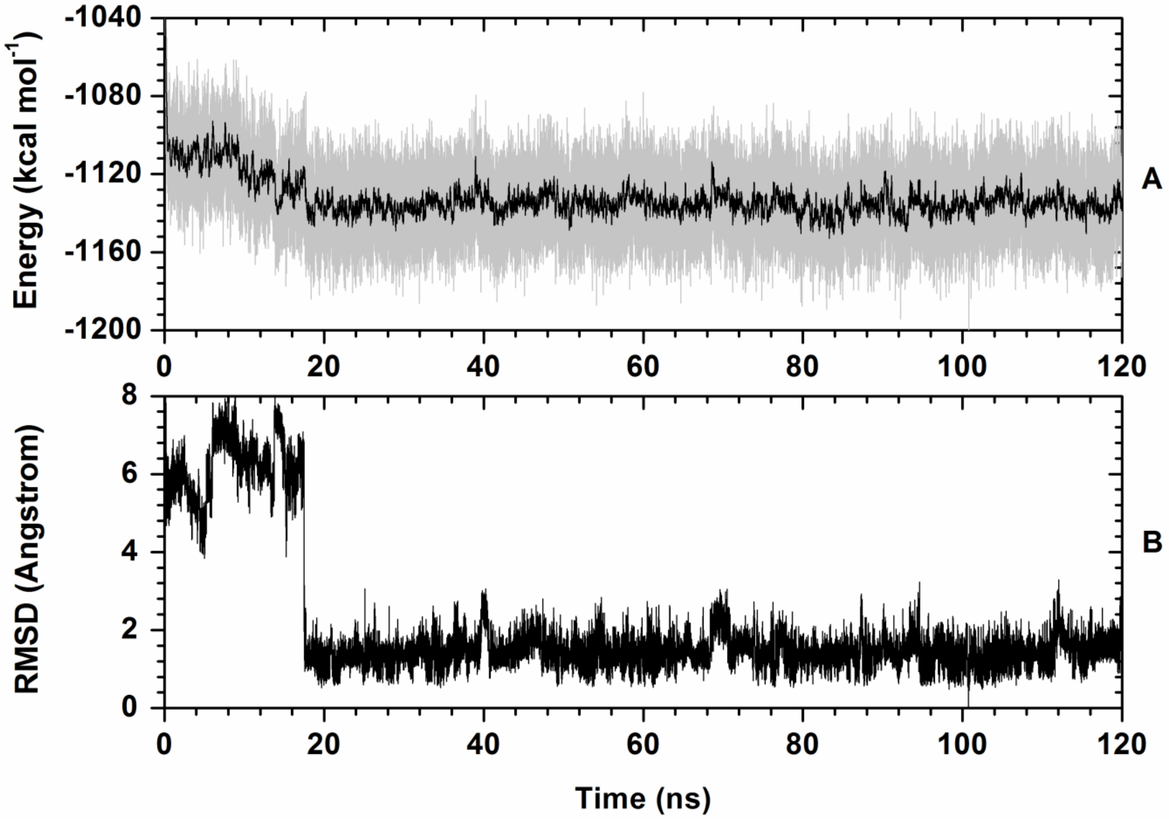

Figure 4 (A) Potential energy of Cgi-ELH2 as a function of time during MD. The solid line is a running average over 100 ps. (B) Backbone rmsd during the same MD, compared to the lowest-energy conformation (the representative structure).

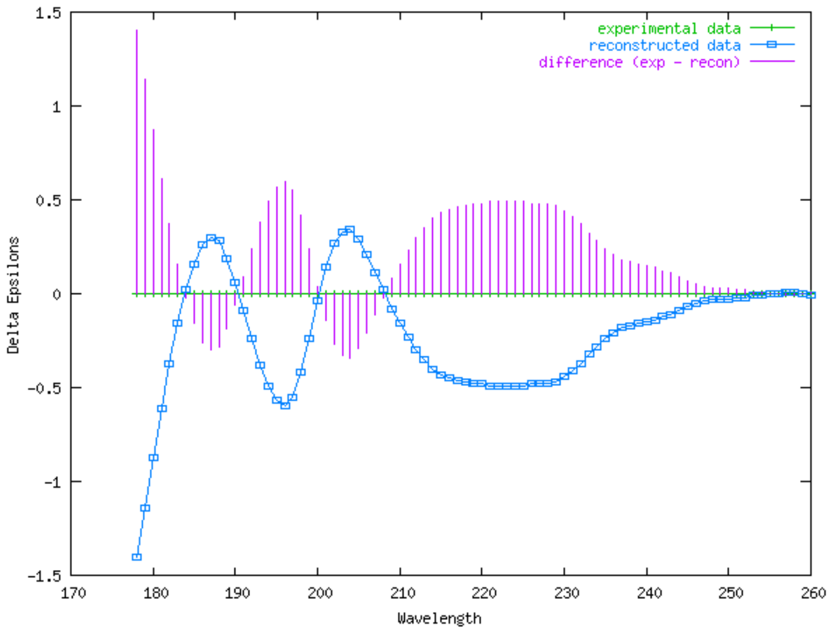

Figure 5. CD spectral analysis of Pf-ELH1.
